# Stress Wave Propagation and Decay Based on Micro-Scale Modelling in the Topology of Polymer Composite with Circular Particles

**DOI:** 10.3390/polym16152189

**Published:** 2024-08-01

**Authors:** Dominik Sabol, Zuzana Murčinková

**Affiliations:** Faculty of Manufacturing Technologies with Seat in Prešov, Technical University of Košice, Bayerova 1, 080 01 Prešov, Slovakia; zuzana.murcinkova@tuke.sk

**Keywords:** decay, explicit FEA, stress wave propagation, ratio, topology

## Abstract

This article deals with stress wave decay performance, analysing the stress wave propagation generated by an impulsive unit load in a 2D representative unit cell (RUC) of a polymer composite with circular particles representing spherical particles, elliptical particles, and short fibres. The micro-scale numerical simulation uses explicit finite element analysis (FEA). The micro-response to an impulsive unit load creates a stress wave amplitude interacting with the material structure and tends to weaken and absorb energy. The stress wave damping is determined by the decaying amplitudes of Mises stress at the front of the stress wave. The stress wave damping is evaluated for different ratios of tensile modules and material densities of matrix and reinforcing material and other factors, such as percentage and particle size, applied to nine topologies of RUCs, and even the presence of an interfacial region is analysed. Moreover, the article visualises the phases of stress wave decay in various particle distributions, i.e., various topologies. Analysing the different topologies of the same particle volume (area) percentage, the study proved that the composite topology and resulting wave–particle and wave–wave interactions are other sources of material damping. The presence of even a small percentage, 3.5 area%, of reinforcing circular particles in the matrix brings a significant increase in stress wave damping up to about 40–43% (depending on the topology) compared to a homogeneous matrix with stress wave damping of 12.5% under the same conditions. Moreover, the topology with the same volume (area) percentage can increase particle stress wave damping by 15.3%.

## 1. Introduction

The internal topology of materials at the meso-/micro-/nanoscopic level influences the macroscopic response in dynamic loading. This applies to all materials, even those that are considered homogeneous in standard calculations, such as steel. The microstructure of steel grains and their mechanical properties affect the macro-mechanical properties as well as other physical properties (more in [1]). The behaviour of technical materials depends on the change in the structural grain size towards nano-areas [2]. Studies of this type date back to the 1970s, as in [3]. In the last decade, there have been many experimental and/or numerical studies focused mainly on stiffness and strength, incorporating the combinations of various parameters and different loading conditions into the studies to achieve advanced behaviour of materials. 

In the presented paper, we wanted to focus on stress wave decay performance and the visualisation of stress wave propagation in dynamic conditions by explicit finite element analysis and stress wave behaviour in different composite topologies, whereas, the volume fraction of particles is not only one factor for the estimation of damping in composites. Moreover, we analysed the other parameters influencing damping. The presented study emphasises that interactions of wave–wave and wave–particle due to topology influence the stress wave damping and are one of the damping sources. 

The internal material damping expresses the natural ability of materials to dissipate mechanical energy. It has been verified that the dissipative energy can be decomposed and associated with the principal stress components [4]. In dynamic conditions, the stress value at a point changes with time, and the stress transmits in the solid. The stress variation in time and space domains is called stress waves [5]. Thus, the connection of stress wave propagation and damping performance is appropriate to determine the topology-related factors based on the unit micro-structure analysis and add them to factors influencing the damping. 

As stated in [6,7,8], the micro-architecture of materials offers the opportunity to obtain unique combinations of material properties. In the last decade, the characteristics of the dynamic behaviour of mechanical systems, such as machine tools, have begun to improve through the utilisation of composite materials with particle structures. With their properties, they contribute to the satisfactory dynamic response of the machine’s operating conditions (more in [9,10,11,12,13,14,15]). Vibration damping in composite materials is very different to metals, and it is a function of two building blocks of the composite system i.e., fibres and matrix, and their respective weight or volume percentages [16]. The selection of the combination of particles and matrix is extremely important because details such as the arrangement, orientation, or aspect ratio of the particles/fibres strongly influence the dynamic behaviour of the material. 

From the mentioned facts, it follows that each composite is a multi-parameter system and the prediction of mechanical properties and their optimisation under given conditions is a very difficult and complex task. For this reason, in studies, only part of the parameters is usually taken into account. Hope is given by [17], which states the necessity of introducing an intelligent platform into the designing process. That platform will integrate technology, including design, manufacturing, monitoring, self-optimisation, and self-healing, during the full life cycle of composites. In this way, the production cost will be much lower in the future, and the performance of production will be enhanced.

Researchers report excellent and promising damping, impact performance, energy dissipation, energy absorbing performance, etc. Aside from toughness, it has been shown that the damping performance can be improved by over 100% with staggered microstructures [18,19]. In [18], it is stated that material architectures give extra control of fracture patterns, improving the critical impact energy by over 6 times. The aluminium foam sandwiches and carbon fibre-reinforced polymer ram showed a damping that is about 20 and 30 times, respectively, greater than the conventional steel ram [9]. The results of [10] showed a high damping ratio of structure filled by particulate polymer composite, which increased the damping of the original structure by 6.5, 40, and 80 times with a polymer composite (replacement of steel) volume fraction 25%, 50%, and 75%, respectively, when it was applied to the grinding machine. In [20], improved damping was obtained by packing machine structures with glass balls, concluding that a packing ratio of approximately 50% is optimal. In [21], it was found that granite material enhanced the dynamic response of the grinding machine structure as compared to the cast iron for modes 3, 4, 5, and 6 by 58.23%, 70.40%, 72.40%, and 56.43%, respectively.

To understand the behaviour of matrix, fibre, and interface when the composite is exposed to static, temperature loading, different characters of dynamic loading, and so on, the finite element method is suitable to use, even in the case of multiscale modelling [22]. Due to some shortcomings of the classical finite element method [23], some authors use explicit finite element analysis (FEA) [24,25], nonlinear finite element analysis [26], and large-scale molecular dynamics simulations, as in [27]. Numerical simulation helps to understand the internal mechanisms of physical–mechanical behaviour and is one of the principal bases in the development of high-damping-capacity materials [16], impact-resistant (protective) materials [27,28], shock-resistant materials, structural barrier materials [27], and ballistic penetration resistance materials [7]. Most of the mentioned research papers are focused on multi-layered materials [27] and sandwich composites, with only a part on hybrids and spherical particles. The presented article focuses on reinforcing particles with a circular cross-section, i.e., spherical, elliptical particles, and short fibres. 

Despite the strong development of computer technologies for modelling and simulating macroscopic models, only a few studies are focusing on investigating the propagation and damping of impulsive, impact, and shock waves, and at the same time their display and visualisation through computational software, which is the focus of the presented article. The shock wave propagation in the statistical volume element of two- and three-phase nanocomposite by elastic FE analysis is analysed and shown in [24]. The compressive shock load response of a cellular glass particle-reinforced thermoplastic composite has been investigated through a mesoscale statistical volume element finite element model in [25]. Computational simulations of the elastic problem of propagation of waves in composite material reinforced by fibres are in [29].

The benefits and novelty of the presented article lie in:

- the method of determining the stress wave damping of the composite material based on the use of explicit FEA analysis of the composite microstructure;
- the quantification of individual parameters affecting the stress wave decay and internal damping of a particulate composite with circular particles;
- the visualisation of the internal damping process using the visualisation of the stress wave in the composite microstructure;
- contribution to the understanding of the physical nature of internal damping.

## 2. Methodology of Simulation of Stress Wave Propagation Generated by Impulsive Load

An ideal elastic wave is a mechanical disturbance that propagates through a material causing oscillations of the particles of that material about their equilibrium positions but no other change [30]. Dissipation mechanisms cause the attenuation of elastic waves. The governing equations of stress waves propagating in three-dimensional space denoted with vector **x** are as follows [31]:

(1) $$\rho \frac{\partial^{2}\Phi (x,t)}{\partial t^{2}}=\left(\lambda +2G\right)\nabla^{2}\Phi (x,t)$$

(2) $$\rho \frac{\partial^{2}\Psi (x,t)}{\partial t^{2}}=G\nabla^{2}\Psi (x,t)$$

where *t* is time, Φ(x,t) is the field of dilatational waves, Ψ(x,t) is a field of shear waves, *ρ* indicates the density of a solid, *λ* and *G* are Lame constants, which describe the linear–elastic region of the solid, and *G* is shear modulus. ∇ and $\nabla^{2}$ are Nabla and Laplacian operators. Because Equations (1) and (2) are wave equations, the solutions can be expressed with velocities *c*_1_ and *c*_2_ as

(3) $$\frac{\partial^{2}\Phi }{\partial t^{2}}=c_{1}^{2}\nabla^{2}\Phi$$

(4) $$\frac{\partial^{2}\Psi }{\partial t^{2}}=c_{2}^{2}\nabla^{2}\Psi$$

where $c_{1}=\sqrt{\left(\lambda +2G\right)/\rho }$ and $c_{2}=\sqrt{G/\rho }$ are velocities of dilatational waves and shear waves.

Based on numerical simulation using explicit FEA, the stress wave damping of particulate composite material is determined in micro-scale analysis. The stress wave damping was determined through the analysis of stress wave propagation and decay of its amplitudes over time as responses to a unit impulsive load. The numerical analysis focused on composites with circular particles. The simulation was aimed at defining and quantifying the parameters significantly influencing the stress wave damping ratio. Analysed parameters are:

- The effect of stiffness and density of particles and the base material (matrix) *E*_m_:*E*_p_ and *ρ*_m_:*ρ*_p_ (*E*_m_ and *E*_p_ is Young’s modulus of matrix and particle, respectively; *ρ*_m_ and *ρ*_p_ is the density of matrix and particle material, respectively);
- The effect of the percentage proportion of particles;
- The effect of the size of particles at their same volume (area) percentage;
- The effect of the topology with circular particles of the composite microstructure.

### 2.1. Computational Model and Numerical Simulation Conditions

The investigation of particle composites at the micro level was carried out using the ABAQUS CAE 2022 software. The matrix of the composite was formed by a material with a density of 1.2 g/cm^3^ and Young’s modulus of 2.4 GPa (Table 1), and the core of the matrix was reinforced with a particle of circular cross-section. The Young’s modulus of the particles in the primary model was 2× larger than that of the matrix.

**Figure 1:**
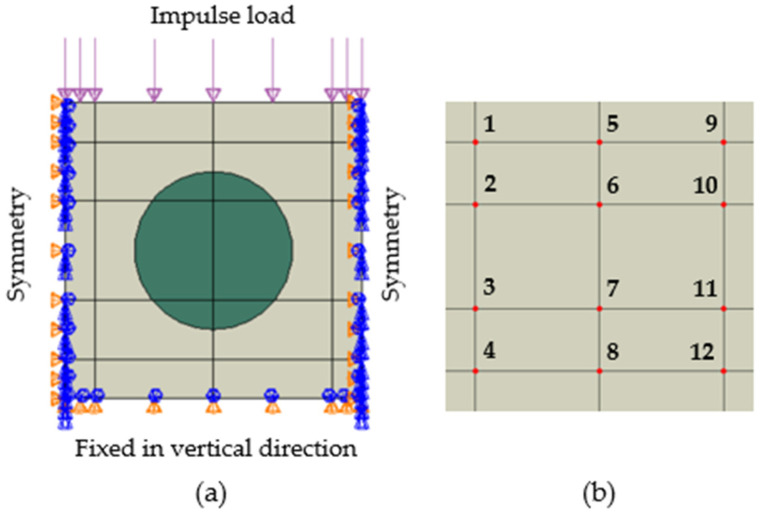
(**a**) Model geometry and boundary conditions, (**b**) layout of points for evaluation.

For analysing the dynamic response to impulsive loading, a representative elementary cell called a representative unit cell (RUC) was selected for the computational model, representing a repeating part of the internal micro-structure of the particle composite (Figure 1a). Thus, the RUC represents an infinite strip in the horizontal direction. RUC is a 2D surface microstructure, and the circular particle in RUC represents the cross-section of the particle, which can be a fibrous, spherical, or elliptical particle.

The assumptions of the computation model were (i) homogeneous, isotropic, and ideal elastic material of the individual phases of the composite, (ii) linear stress–strain response range (elastic wave), (iii) perfect adhesion between the particles and the matrix, i.e., no inter-domain displacements, and (iv) no residual stress in RUC.

The impulse pressure load was applied perpendicularly, on the top edge of the numerical model (Figure 1a) with a maximum value of 10MPa increasing from zero to its maximum value with a drop back to zero in 2 × 10^−7^ s. Thus, the load is not static but dynamic corresponding with the bump of a hammer. The displacement in the vertical direction was fixed at the bottom edge. Symmetry conditions are defined on both vertical edges (Figure 1a).

Figure 1b shows the identification points that are part of the model and at which the Mises stress values are computed as a function of time during the propagation of the stress wave. The time interval 0 ÷ 1 × 10^−5^ s was specified to determine the time response of the stress wave. 

**Figure 2:**
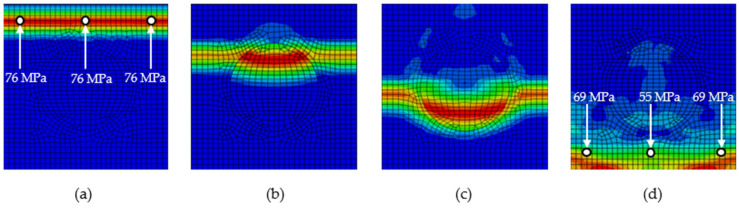
Phases of stress wave spread in RUC (**a**) phase 1, (**b**) phase 2, (**c**) phase 3, (**d**) phase 4.

**Figure 3:**
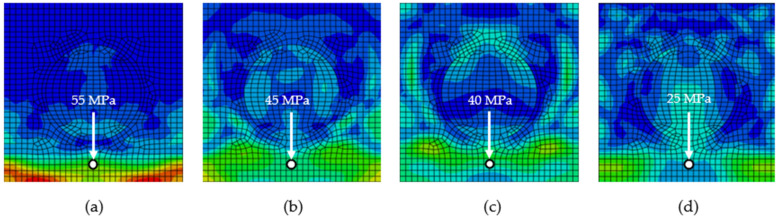
Stress wave at time *t* = 10^−5^ s in numerical models with ratio *ρ*_m_:*ρ*_p_ 1:1 and *E*_m_:*E*_p_ (**a**) 1:2, (**b**) 1:5, (**c**) 1:10, (**d**) 1:100.

The finite element mesh consists of CPS4R elements that are 4-node bilinear planar quadrilaterals with reduced integration. The area of one finite element was about 0.03% of the total simulated RUC area. The density of the mesh was determined with a minimum curvature factor of 0.1 (10%) and minimum size factor 0.1 (10%). The number of finite elements was 1178 in Figure 2 and Figure 3, and 2916, 3027, 3132, 3179, 3217, 3574, 3579, 3127, 4011, and 3973 for topologies A–I. The finite element meshes are visible in Figure 2, Figure 3, Figure 8 and Figure 10.

### 2.2. Evaluation of Numerical Results

**Figure 4:**
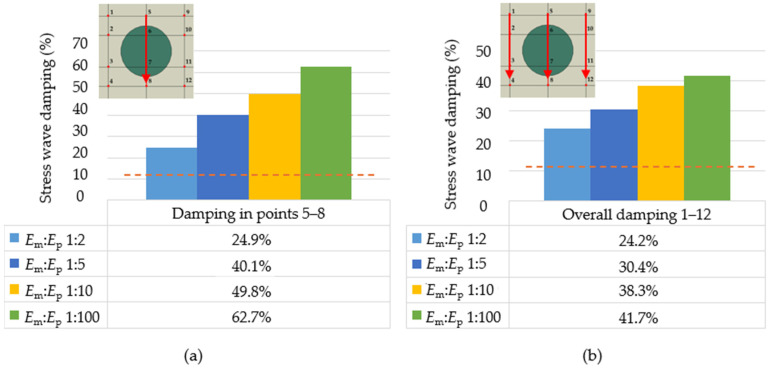
Decay of Mises stress at *ρ*_m_:*ρ*_p_ 1:2 and different *E*_m_:*E*_p_ (**a**) evaluated from one centre line of points 5–8, (**b**) evaluated from three lines of points 1–4, 5–8, and 9–12, i.e., overall stress wave damping; orange dashed line is for a model without particle indication, with an overall stress wave damping of 12.5%.

Applying an impulse load, a stress wave is created in the RUC, starting to propagate from the upper edge of the RUC. The stress wave gradually propagates throughout the composite structure, and its values decrease. The stress wave damping was defined as the decrease in the Mises stress in the three lines of identification points, as shown in Figure 1b and Figure 4, i.e., points 1–4, 5–8, and 9–12, in all modelled topologies of the particle composite. The stress wave damping *D*_SW_ in lines of identification points 1–4, 5–8, and 9–12 was calculated as:

(5) $$D_{\mathrm{SW}}=\frac{\sigma_{Mi}-\sigma_{Mi+3}}{\sigma_{Mi}}\times 100\%;\;\;\;i=1,5,9$$

where *σ*_M*i*_ is the value of the Mises stress at points 1, 5, and 9, and *σ*_M*i*+3_ is the value of the Mises stress at points 4, 8, and 12. The values *σ*_M_ are the values of the first maximum Mises stress amplitudes of the propagating stress wave, i.e., the values at the front of the stress wave.

## 3. Results

### 3.1. Numerical Simulation of Different Stiffness and Density Ratios of Matrix and Particle

An impact applied to the upper edge of the particle composite causes a stress wave to propagate vertically down the full width of the model. (Figure 2a). Initially (phase 1, Figure 2a) the propagation of the stress wave is in a homogeneous matrix without any interaction with the particle. This homogeneous stress wave would propagate as a homogeneous wave in a base material without a particle. However, in the next phase (phase 2, Figure 2b) the front of the stress wave gradually interacts with the stiffer particle, which causes the weakening of the stress wave (phase 3, Figure 2c), i.e., the stress values are decreased. By gradually passing through other parts of the model, the Mises stress of the last identification points is the lowest (phase 4, Figure 2d). The values of the Mises stress in Figure 2 are for *E*_m_:*E*_p_ 1:2 and *ρ*_m_:*ρ*_p_ 1:1.

To compare the effect of particle stiffness against impulse loading, simulations were performed on models with a ratio of *E*_m_:*E*_p_ 1:2, 1:5, 1:10, and 1:100. Figure 3 shows the stress wave corresponding to phase 4 from Figure 2, i.e., at time *t* = 1 × 10^−5^ s. It is possible to compare the values of the Mises stress at the highlighted point 8 from Figure 1b. The increasing stiffness of the particle has a decreasing effect on the Mises stress at the monitored point 8.

To compare the method of evaluation, the stress wave damping was determined (i) from the values at the front of the stress wave in one centre line, i.e., between points 5–8 and (ii) as an average value of three lines of points 1–4, 5–8, and 9–12, according to relation (5) (Figure 4). The values in Figure 4 are the overall stress wave decay values, which are 0.7 (24.9–24.2%); 9.7, 11.5, and 21.0% (on average by 10.7%) smaller than those from only one centre line 5–8. In the next sections of the article, the stress wave damping will be evaluated from all three lines of points, i.e., overall stress wave damping is evaluated.

The RUC with only the matrix, i.e., without the particle, has an overall stress wave damping value of 12.5% (dashed orange line in Figure 4). The effect of the presence of the particle on the stress wave damping is significant, representing an increase of 11.7 (24.2–12.5%), 17.9, 25.8, and 29.2% for the corresponding stiffness ratios *E*_m_:*E*_p_ and *ρ*_m_:*ρ*_p_ 1:2. From the obtained results, it can be concluded that if the stiffness of the particle increases, the stress wave damping is larger.

**Figure 5:**
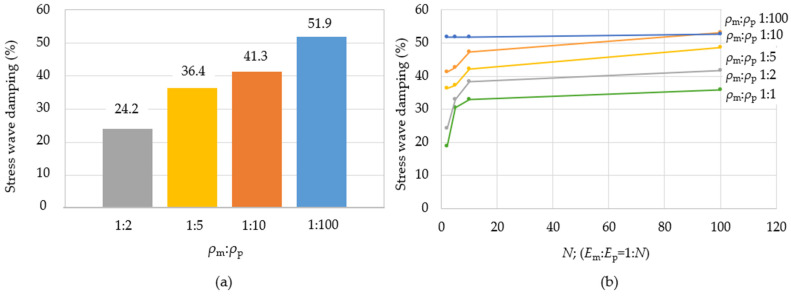
Decay of the Mises stress (**a**) at *E*_m_:*E*_p_ 1:2 and different *ρ*_m_:*ρ*_p_ composite structures, (**b**) effect of stress wave damping of different *E*_m_:*E*_p_ and *ρ*_m_:*ρ*_p_.

Furthermore, the different density ratios of the matrix to particles *ρ*_m_:*ρ*_p_ 1:2, 1:5, 1:10, and 1:100 were analysed for the numerical model, while *E*_m_:*E*_p_ is 1:2. The geometry and boundary conditions were the same as described in Figure 1a. However, the computation time was increased from 1 × 10^−5^ s to 1.3 × 10^−5^ s due to the more difficult propagation of the stress wave through the denser particle material. The stress wave damping behaviour had a similar character to the change in Young’s modulus ratio, and thus, by increasing the particle density, the damping of the stress wave also increased, i.e., values of the stress wave are decreased. The largest stress wave damping occurred at *ρ*_m_:*ρ*_p_ 1:100, with 51.9% damping (Figure 5a). The stress wave damping values at the 1:2 ratio were about twice as low as values at the 1:100 ratio. From this point of view, it can be concluded that the higher the ratio of the density of the reinforcing particles to the matrix, the larger the damping of the stress wave. In Figure 5b, the summary of the effects of ratios *E*_m_:*E*_p_ and *ρ*_m_:*ρ*_p_ on the stress wave damping is given. The most increased values of the stress wave damping are in the range *E*_m_:*E*_p_ 1:2–1:10.

### 3.2. Numerical Simulation of Different RUC Topologies 

**Figure 6:**
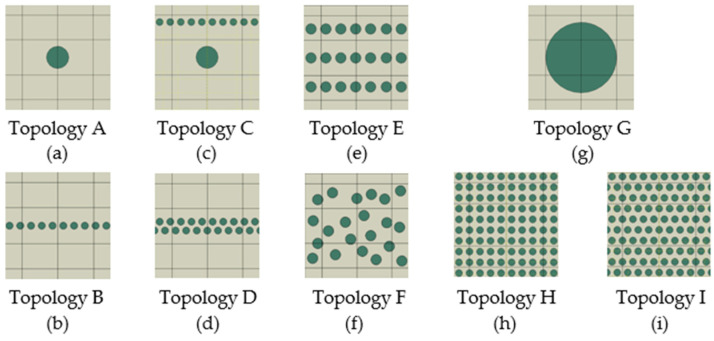
Topologies of RUCs: (**a**,**b**) RUCs of 3.5 area%, (**c**,**d**) RUCs of 7.0 area%, (**e**,**f**) RUCs of 16.5 area %, and (**g**–**i**) RUCs of 35.0 area%.

The individual topologies of RUCs differed in arrangement, particle area percentage, and particle size (Figure 6). Moreover, the different topologies with the same particle area percentage were compared. Individual topologies of computational models (Figure 6) contained 3.5%, 7.0%, 16.5%, and 35.0% particle area percentage of RUC, representing a repeated microscale structure. The particle percentage is expressed by area, as the numerical model of RUC is 2D. Recall that the circular particle/s in RUC represents the cross-section/s of fibre/s or spherical or elliptical particle/s. The computation conditions, including time interval and boundary conditions, were retained, as described in Section 2 and Figure 1. Similarly, the ratio of Young’s modulus *E*_m_:*E*_p_ 1:2 and density *ρ*_m_:*ρ*_p_ 1:2 was the same in all topology models shown in Figure 6. The method of evaluating the numerical results was the same as in previous numerical models.

**Figure 7:**
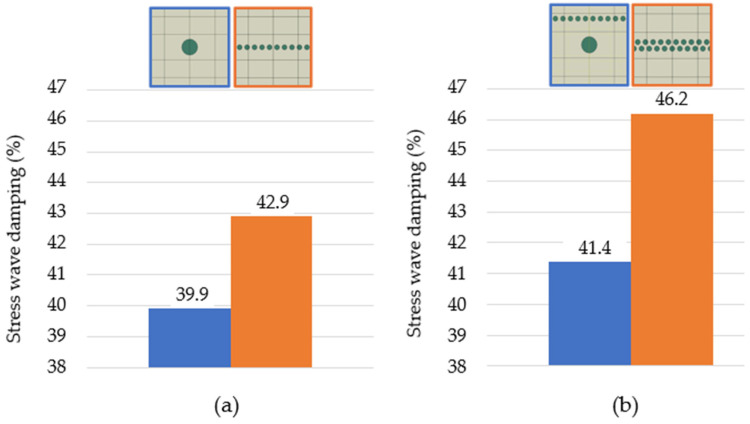
Stress wave damping values for the RUC topologies: (**a**) of 3.5% and (**b**) of 7.0% particle area percentage.

**Figure 8:**
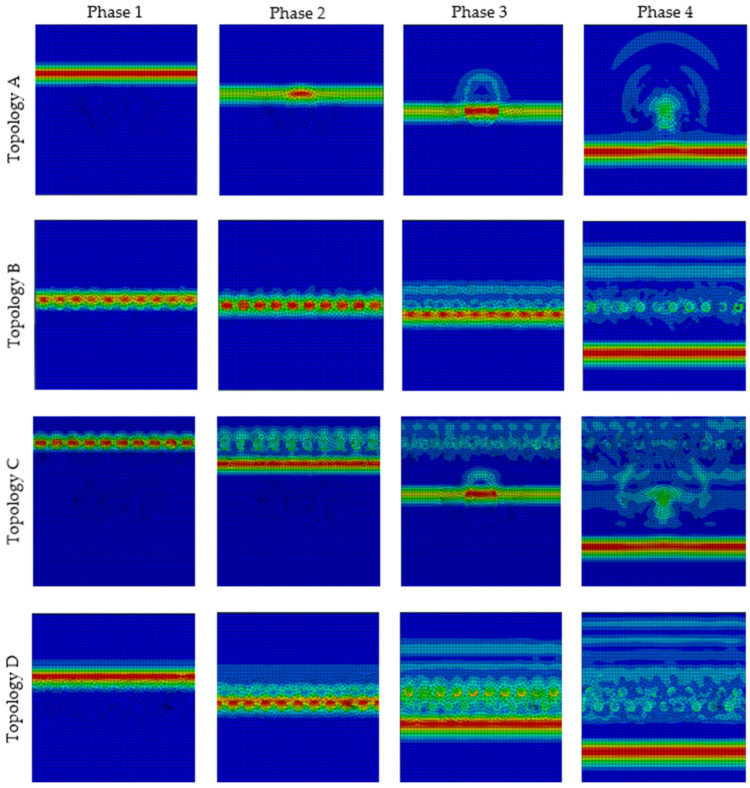
Visualisation of stress wave propagation phases for RUCs topologies A–D.

Two topologies of composite structures with a 3.5% particle area percentage were compared, the topology with one large particle (Topology A, Figure 6) and the topology with 10 small particles arranged in one row (Topology B, Figure 6). Topology B of the composite structure with 10 smaller particles achieved stress wave damping 3% greater (Figure 7a) than that of topology A, i.e., structure with 1 particle. The source of the greater stress wave damping is due to multiple interactions, which include the multiple reflections and refraction of the stress wave on the stiffer and smaller particles. The phases of the stress wave propagation and the mentioned phenomena can be seen and compared in Figure 8.

Analysing the composite structures with the same 7.0% area fraction of particles in RUC, another two topologies were compared. Topology C (Figure 6) consisted of a large particle located in the centre and together with 10 smaller particles in one row located in the upper part of the RUC. Topology D (Figure 6) consisted of 20 particles in two rows in the centre of the RUC, while the second row was shifted between the gaps of the first row particles. The stress wave damping results (Figure 7b) indicate that Topology D with smaller and overlapped particles located in the centre of the matrix had a more effective stress wave damping, namely greater by 4.8%. 

In Figure 7, it can be seen that a larger percentage fraction of particles increases the stress wave damping, which is the expected result. However, it is necessary to emphasise that the effectiveness of particle presence and influence on stress wave damping for the same particle area fraction can be increased by topology (particle arrangement) and particle size. RUCs of different topologies A and B and the topologies C and D have the same particle area percentage (3.5% and 7.0%). However, different topologies changed stress wave damping by 3.0 and 4.8%, respectively.

Figure 8 shows four phases of the stress wave. The individual phases are shown at different times. The front of the stress wave has the greatest amplitude at a given moment and is shown in red. In Figure 8, the stress wave propagation phases show that the presence of circular and elliptical particles and short fibres affects the increase in interaction of wave–particle and wave–wave, resulting in reduced amplitudes, increased stress wave damping, and more efficient absorption of wave energy compared to a homogeneous material. The interactions of the stress wave with the particles and the mutual interactions of the waves during wave propagation cause multiple increases in phenomena such as absorption, reflection, diffraction, and refraction. Mainly in phase 4, the back reflection of the stress wave can be observed, increasing the weakening of the amplitude and energy of the forward stress wave.

In real composite materials, the arrangement of reinforcing particles is mostly random for technological reasons, so we compared this arrangement with a regular arrangement (Topology E and F, Figure 6). These numerical models contained 21 particles with the same area fraction of 16.5 area%. All computational conditions, i.e., impulse loading and boundary conditions, were retained as described in Section 2 and Figure 1. Likewise, the ratios of Young’s modulus *E*_m_:*E*_p_ and density *ρ*_m_:*ρ*_p_ 1:2 are the same as in the previous models. The simulation was computed in 120 cycles of a uniformly distributed time interval of 2.1 × 10^−5^ s at a time step of 1 × 10^−7^ s. The damping of the stress wave after impulse loading was evaluated in the same way as in the previous numerical models. Comparing the uniform arrangement of particles (46.7% for topology E) and a random geometric distribution of particles (50.4% for topology F), the stress wave damping is greater by 3.7%. The above-mentioned stress wave damping values are the numbers that can be varied over a small range depending on the possible larger or smaller clustering of randomly arranged particles concerning the direction of particle propagation. 

**Figure 9:**
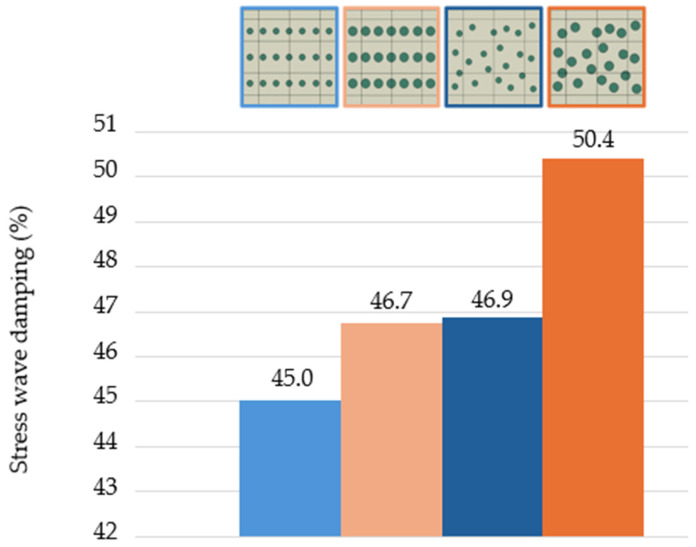
Comparison stress wave damping for regular and random arrangements and different particle sizes.

The determined stress wave damping values in Figure 9 correspond with the above-mentioned conclusions. Figure 9 compares the stress wave damping for regular and random distribution at the same and different particle percentages in identical arrangements. The random arrangement at the same particle percentage slightly increases the stress wave damping. Moreover, comparing the different particle percentages (7.3 area% and 16.5 area%), the larger the particle percentage, the larger the stress wave damping at regular and also at random arrangement.

**Figure 10:**
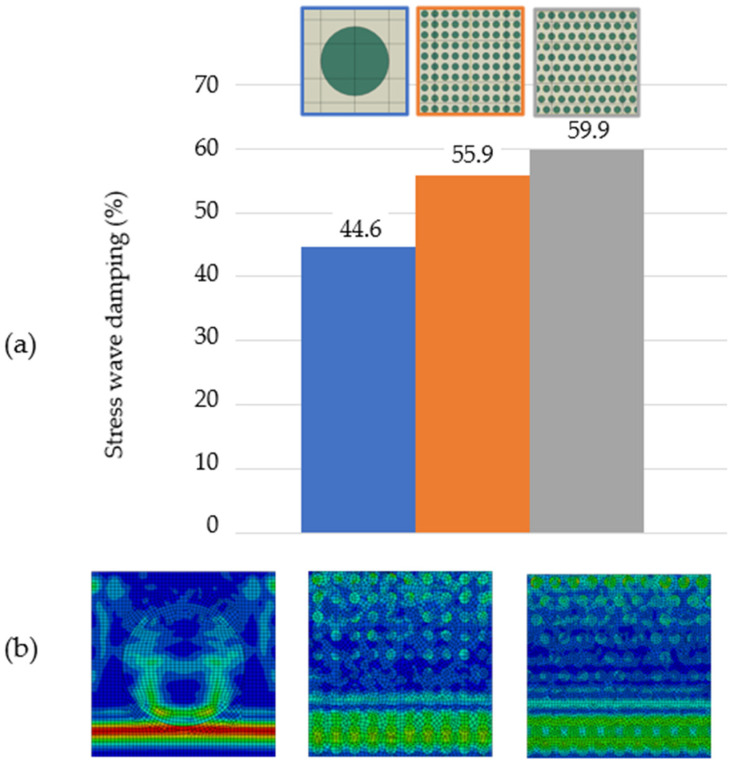
Composite structures with RUCs of 35 area% topologies G, H, and I: (**a**) stress wave damping and (**b**) the last phase of stress wave propagation.

Three topologies of composite structures were compared, analysing the 35.0 area%. Topology G (Figure 6) is reinforced with one large particle located at the centre of the matrix, topology H is reinforced with 10 particles in rows of 10 particles distributed in RUC without overlap in topology H and with an overlap, i.e., every second row is shifted between the gap of the previous row, in topology I. The highest stress wave damping, almost 60% (Figure 10), was for small overlapping particles in topology I. This stress wave damping was also the highest among all topologies A–I of RUCs. Figure 10b shows the last-phase images of the stress wave propagation. 

The dispersion of the stress wave as well as its energy is higher for the topology with small particles at the same particle fraction, which is in the context of the previous conclusions, with an advantage for overlapping rows. The overlapping rows of particles increase the amount of wave–particle and wave–wave interactions as it was described in Figure 8 resulting in a higher stress wave damping.

## 4. Discussion

In this paper, we have identified and analysed the parameters that will be considered significant parameters for the intended optimisation study to maximise stress wave damping. In addition to the geometrical arrangement of the particles, the selection of material components is very important, which contributes to the stress wave damping efficiency by their material properties, such as stiffness and density. 

Numerical models showed an advantage for small particle sizes at the same percentage fraction of particles in the matrix. A higher number of small particles means a larger length (area) of the interface. In our case, comparing the same particle percentage in Topology A and B, a 3.2-times longer interface caused an increase in stress wave damping by 3%. In Topology G, H, and I, a 10-times longer interface caused an increase in stress wave damping by 11.3% to 15.3%, depending on particle distribution. There is an obvious correlation between these values of interface size and stress wave damping. Similar results are reported in [32,33,34]. Moreover, in [32], an additive or filler that is either viscoelastic or small in unit size is attractive. The above correlation is related to interfacial interactions leading to enhanced damping capacity. However, in the models presented in this article, the matrix–particle connection is fixed, which does not allow slippage, so it can be assumed that due to slippage at interface, the real damping is higher than the calculated one. The source of stress wave damping in the presented models is the interaction of the stress wave with particles (wave-particle) and the mutual interaction of waves (wave–wave) as well as the reflection, refraction, and diffraction of the stress wave.

Of course, increasing the percentage of particles improves the stress wave damping, as seen on presented models from 3.5 area% to 35.0 area%. It means that more particles increase the size of the interfacial interactions and the interactions of the stress wave, which is energy-consuming. Moreover, by changing the particle distribution, i.e., changing the topology, at the same percentage fraction, the stress wave damping can be increased considerably, by 15.3% (Figure 10). A 35 area% of particles and their arrangement increased the stress wave damping to 60%. 

**Figure 11:**
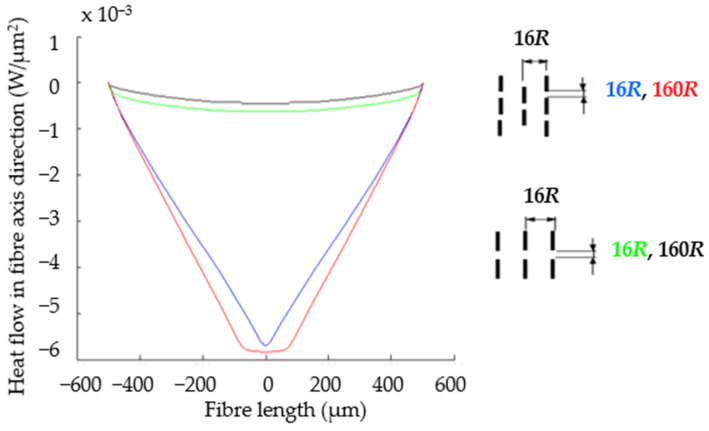
Analysis of fibre gap spacing through heat flow in fibres with *L*:*R* = 1000 and constant axis distance.

In [35], it is stated that spherical particles have the best damping properties, i.e., fibres with a ratio of *L*/*D* = 1:1. In fibre composites, the fibre end-gap spacing becomes important. The composite damping generally increases with increasing fibre end gap size because of the increased amount of matrix material between abutting fibre ends [35]. The analysis of the space gap between the ends of the fibres and their axial distance is in [36]. Through the analysis of heat flow of overlapping and non-overlapping fibres in a group of fibres with different aspect ratios *L*:*R*, the thermal conductivity of the fibres is higher than the matrix. Fibres with overlap (blue and red) show 11.5 times greater heat flow in the fibre direction (Figure 11) than fibres without overlap (black and green). The overlapping fibres are adjacent to the gap and “help” to increase the heat flow. Analogously to the above, it also applies to the axis force or normal stress.

**Figure 12:**
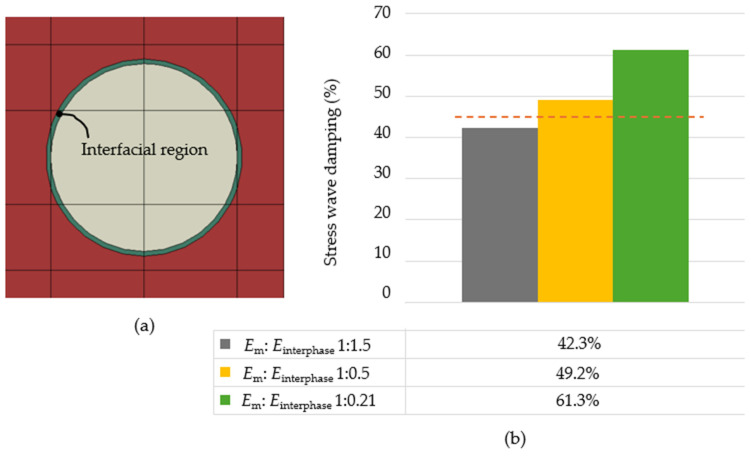
Topology G (*E*_m_ = 2.4 GPa, *E*_p_ = 4.8 GPa, i.e., 1:2, *ρ*_m_:*ρ*_p_ 1:2) with interfacial region: (**a**) numerical model, (**b**) the results summary.

The factor improving damping of composites is also the third phase of the composite (interfacial region), as stated in [31,32,33,34,35,37]. The stress wave damping without the interfacial region for the given topology G was 44.6% (orange dashed line in Figure 12b). The material of the interfacial region that is stiffer than that of the matrix, i.e., *E*_m_ = 2.4 GPa and *E*_interphase_ = 3.6 GPa, ratio 1:1.5, worsened the stress wave damping. Advantageously, in terms of stress wave damping, the interface should be less stiff than the matrix. For example, for 0.21 times smaller *E*_interphase_ than Young’s modulus of the matrix (*E*_m_:*E*_interphase_ = 1:0.21) the stress wave damping increased by 16.7%.

We can summarise that topology-related factors are: (i) wave–particle interaction, (ii) wave–wave interaction, (iii) matrix–particle interface size, and (iv) matrix–particle interface character, e.g., its stiffness, adhesion, friction. The mentioned factors result in increasing the stress wave propagation time. 

## 5. Conclusions

We contributed to understanding the physical nature of damping due to microarchitecture topology. In dynamic responses of inhomogeneous materials, such as composites including, e.g., reinforcement elements, wave propagation is still a subject of research to describe the dynamic response process at the microscale. Propagation of an impulse wave in composite material components can be characterised as a complicated matter from the point of view of its dispersion behaviour. An impulse wave in a pure homogeneous material is “homogeneous”, and its character changes only at the boundaries of the investigated model. However, in a composite material with reinforced particles, there is a complicated interaction of the impulse wave, with reflection and refraction already at the interface between the softer matrix and the stiffer particles, which causes very effective dispersion and damping. The article analysed sources of stress wave damping such as the ratios between Young’s modules and the densities of the matrix and particles of polymer composite, with circular particles representing spherical, elliptical, and short fibres, and the dependence of stress wave damping on the topology of the RUC composite. The properties of the material and its specific structure (topology) can significantly affect the propagation of stress waves.

From the results of the numerical simulation, it can be clearly stated that the larger the Young’s modulus of the circular particles and the density of the circular particles, the greater the damping of the stress wave after impulse loading, while the most significant increase is in the range of *E*_m_:*E*_p_ 1:2–1:10. Further, already a small percentage of circular particles (3.5 area%) of reinforcing particles in the matrix brings a significant increase in stress wave damping values compared to homogeneous materials with 12.5% stress wave damping at the same conditions. The particle distribution, i.e., topology, is also an important factor in addition to other factors, such as the ratios *E*_m_:*E*_p_ and *ρ*_m_:*ρ*_p_, the particle size, and particle percentage. With the same percentage fraction, by changing the topology, the increase in the stress wave damping by 15.3% was achieved. For example, for 35 area% particles in RUC, and by changing the topology, it was possible to increase the stress wave damping to 60%.

In the Discussion section, the article points to and analyses another source of stress wave damping, namely, the influence of the interfacial region and its length and stiffness relative to the matrix and the particle. This third composite phase represents another beneficial contribution to stress wave damping performance and is suitable for further research.

The mentioned analysis of stress wave damping sources identifies individual factors, quantifies them, and presents a sensitive study of these factors. It visualises the phases of the stress wave decay in the microscale, and in this way, it is possible to visualise the wave–particle, and wave–wave interactions, which are important for the absorption of the excitation energy and the subsequent effective damping.

It can be assessed that the implementation of composite structures, in the form of particle composites for vibration damping, represents a promising area for significant improvements in performance and safety in different industries.

## Figures and Tables

**Table 1:** Mechanical properties of the matrix and particle materials.

	Young’s Modulus *E* (GPa)	*Ratio* *E*_m_:*E*_p_	Poisson’s Ratio *ν*	Density *ρ* (g/cm^3^)	*Ratio* *ρ*_m_:*ρ*_p_
Matrix	*E*_m_ = 2.4 GPa	-	0.35	*ρ*_m_ = 1.2 g/cm^3^	-
Particle	2.4 × 1	1:1	0.35	1.2 × 1	1:1
	2.4 × 2	1:2		1.2 × 2	1:2
	2.4 × 5	1:5		1.2 × 5	1:5
	2.4 × 10	1:10		1.2 × 10	1:10
	2.4 × 100	1:100		1.2 × 100	1:100

Note: The lower indexes “m” and “p” are for matrix and particle material, respectively.

## Data Availability

Data are contained within the article.
